# Revisiting Aurochs Haplogroup C: Paleogenomic Perspectives from Northeastern China

**DOI:** 10.3390/genes16060639

**Published:** 2025-05-27

**Authors:** Yan Zhu, Xindong Hou, Jian Zhao, Bo Xiao, Shiwen Song, Xinzhe Zou, Sizhao Liu, Michael Hofreiter, Xulong Lai

**Affiliations:** 1 School of Environmental Studies, China University of Geosciences, Wuhan 430078, China; 2 State Key Laboratory of Geomicrobiology and Environmental Changes, China University of Geosciences, Wuhan 430078, China; 3 Department of Scientific Research, Dalian Natural History Museum, Dalian 116023, China; 4 Institute for Biochemistry and Biology, University of Potsdam, Karl-Liebknecht-Strasse 24-25, 14476 Potsdam, Germany; 5 School of Earth Sciences, China University of Geosciences, Wuhan 430074, China

**Keywords:** aurochs, paleogenomes, phylogeny, divergence time, population dynamics

## Abstract

Background/Objectives: Aurochs (*Bos primigenius*), one of the earliest and largest herbivores domesticated by humans, were widely distributed in Eurasia and North Africa during the Pleistocene and Holocene. Studies of aurochs in China have focused mainly on the Northeastern region. Previous studies have suggested that haplogroup C is a haplogroup unique to China, but recent studies have shown that this is not the case. We have compiled all data on haplogroup C to revisit the classification of the aurochs haplogroup C. Methods: In this study, we obtained 13 nearly complete mitochondrial genomes from Late Pleistocene to early Holocene bovine samples from Northeastern China through fossil sample collection, ancient DNA extraction, library construction, and high-throughput sequencing. Based on the acquired ancient DNA data and in combination with previously published bovine data, the phylogenetic status, lineage divergence time, and population dynamics of aurochs in Northeastern China were analyzed. Results: Phylogenetic analyses and divergence time estimations suggest that the current definition of haplogroup C is overly inclusive, necessitating a refined reclassification of this haplogroup. We also estimated the population dynamics of aurochs in Northeastern China using Bayesian skyline plots found that the maternal effective population size of the aurochs increased significantly during Marine Isotope Stage 5 (MIS5), but began to decrease in the second half of MIS3 before they eventually became extinct. Conclusions: Our results provide new molecular evidence on the phylogenetic status, divergence time, and population dynamics of aurochs in Northeastern China.

## 1. Introduction

The auroch (*B.primigenius*) is the ancestor of one of the most important livestock species, the domesticated cattle, and was widespread in Eurasia and North Africa during the Pleistocene and Holocene [1,2,3]. Aurochs have been geographically classified into four subspecies: the Eurasian subspecies (*Bos primigenius primigenius*), the Indian subspecies (*Bos primigenius namadicus*), the North African subspecies (*Bos primigenius mauretanicus*), and the Chinese subspecies (*Bos primigenius suxianensis*) [1]. Aurochs primarily inhabited enclosed forests, marshy areas, and fluvio-lacustrine environments [4]. Their diet consisted mainly of grasses, supplemented by forbs, leaves, and branches of trees and bushes [5]. Due to climatic oscillations and human hunting, the aurochs population declined over millennia and became finally extinct in the 17th century, with the last documented female aurochs dying in Poland in 1627 [6].

The fossil record of aurochs in Europe can be traced back to the late Middle Pleistocene (0.5–0.6 million years ago, Ma) [7,8]. The historical range of aurochs in North Africa extends from the early Middle Pleistocene to 2800 BC [9,10]. Aurochs in China appeared in either the Middle or Late Pleistocene [4]. In China, aurochs remains are predominantly distributed across the Northern, Northeastern, and Shaanxi–Gansu regions, with fewer discoveries documented in Central Southern, Southwestern, and Eastern China [4]. Chronologically, Northern Chinese auroch specimens date earlier, spanning from the Middle Pleistocene to approximately 3500 years before present [11], while those from the Northeastern region are generally later, predominantly belonging to the Late Pleistocene, with the youngest remains dated to around 1100 BCE [12].

In recent years, much molecular research on aurochs has been carried out worldwide. Previous studies initially identified six mitochondrial haplogroups in aurochs, designated as haplogroups P, T, E, Q, R, and I [4,7]. Subsequently, Zhang et al. (2013) described a haplogroup C, based on a Late Pleistocene auroch specimen from Northeastern China [13]. Haplogroups T and P are commonly found in Eurasian aurochs, while haplogroup E has only been identified in Neolithic samples from the Eilsleben site in Germany [7]. In contrast, haplogroups Q and R are relatively rare [14]. Initially, haplogroup C was reported exclusively in Northeast Asia [4,13,15,16,17]. However, recent research has demonstrated the presence of haplogroup C in Russia and Kazakhstan, alongside the discovery of two additional haplogroups, K and G, in Europe and Central Asia. Notably, haplogroup G occupies a basal position in the phylogeny of aurochs and domestic cattle [18]. Divergence time estimates for haplogroups P, Q, T, and I were provided by Achilli et al. (2008) [19]. Hou et al. (2024) estimated the divergence of haplogroup C from its sister lineage to have happened approximately 150 thousand years ago (Ka) and its most recent common ancestor (tMRCA) to about 90 Ka [15,19]. In contrast, Rossi et al. (2024) suggested a younger divergence time (~77 Ka) and tMRCA for this haplogroup (~60 Ka) [18]. In conclusion, with the discovery of more samples, the distribution range and evolutionary time frame of each haplogroup are still changing.

Contemporary molecular investigations on Chinese aurochs have predominantly concentrated on the Northeastern region of China, where the majority of analyzed specimens belong to haplogroup C. The predominance of this genetic lineage within the regional population establishes this geographical region as a pivotal zone for investigating aurochs’ evolutionary history and population dynamics. In this study, we utilized 13 mitochondrial genome sequences obtained from *Bos* samples from Northeastern China, combined with previously published data, to explore the phylogenetic position of aurochs in Northeastern China, estimate the divergence time of different haplogroups, and raise questions about the current classification of haplogroup C. At the same time, a Bayesian skyline analysis was used to reconstruct the population dynamics of aurochs in Northeastern China, providing new molecular evidence for studying the population history and extinction reasons of aurochs in Northeastern China.

## 2. Materials and Methods

### 2.1. Sampling Information

A total of 13 *Bos* specimens from Northeastern China were collected in this study, including 11 samples that were excavated from Zhaodong, and two samples from Qinggang, Heilongjiang Province (Figure S1 and Table S1). Six of the samples have been subjected to accelerator mass spectrometry (AMS) radiocarbon dating at β Analytic Testing Laboratory in the USA and calibrated using the IntCal20 curve.

### 2.2. DNA Extraction, Double-Stranded Library Construction, and Sequencing

We performed DNA extraction and double-stranded library preparation in a specialized ancient DNA laboratory at the China University of Geosciences (Wuhan). Each sample was ground into powder, weighed about 150 mg, mixed with 3 mL of EDTA (0.5 M, pH 8.0) and 40 µL of Proteinase K (20 mg/mL), and incubated overnight at 37 °C under constant agitation. After centrifugation at 7000 rpm for 10 min, the supernatant was added to an ultrafiltration tube (Millipore, Darmstadt, Germany) and was condensed to about 100 µL at 7000 rpm for 35 min. Then, the DNA was purified using the MinElute PCR purification kit (Qiagen, Hilden, Germany) and eluted twice with 50 µL EB buffer according to the manufacturer’s instructions. To monitor potential contamination, blank controls were set up in all experimental steps.

Ancient DNA libraries were constructed using 20 µL DNA extract according to the protocol of Meyer and Kircher [20]. In the blunt-end repair step, DNA templates were mixed with NEB buffer 2 and BSA (New England Biolabs, Ipswich, UK), and then DNA was purified using the MinElute PCR purification kit (Qiagen, Hilden, Germany). A 1:20 adapter diluted with Quick Ligase buffer (New England Biolabs, Ipswich, UK) was mixed with templates in the Adapter Ligation step. The Isothermal buffer (New England Biolabs, Ipswich, UK) was used in Adapter Fill-in. The indexing amplification of the library used Q5 Hot Start High-Fidelity 2 × Master Mix with amplification conditions of 95 °C for 2 min, 95 °C for 15 s, 60 °C for 30 s, and 68 °C for 30 s, with 17 cycles. Library concentration and fragment size were measured using Qubit 4.0 (Invitrogen, Carlsbad, CA, USA) and TapeStation 4150 (Agilent, Santa Clara, CA, USA). Libraries were sequenced on an Illumina NovaSeq6000 platform at Annoroad Inc., Beijing, China.

### 2.3. Single-Stranded Library Construction and Hybridization Capture

DNA extraction from three ancient samples (CADG442, CADG444 and CADG445) was conducted in a dedicated ancient DNA laboratory at the University of Potsdam, following the silica-based purification protocol of Sheng et al. [21]. Single-stranded DNA libraries were constructed from 20 μL of extract using the Gansauge and Meyer method [22], with modifications for uracil removal as described in Yuan et al. [23]. For mitochondrial genome enrichment, hybridization capture was performed according to González Fortes and Paijmans [24], utilizing biotinylated bait generated from modern cattle DNA: Muscle-derived genomic DNA (DNeasy Blood & Tissue Kit, Qiagen, Hilden, Germany) was amplified via three overlapping LR-PCR primer pairs spanning the mitochondrial genome. The resulting amplicons were fragmented, blunt-end repaired, and ligated to biotinylated adapters. Two iterative rounds of capture were applied to maximize efficiency. Equimolar pooled libraries were sequenced on an Illumina NextSeq 500 platform in 75 bp single-end runs using the method of Paijmans et al. [25].

### 2.4. Data Processing

Raw sequencing reads were subjected to adapter trimming and stringent quality filtering using fastp-0.22.0 [26]. Reads shorter than 30 bp or with average Phred scores < 20 were discarded to minimize low-confidence alignments. Filtered reads were mapped to the taurine cattle mitochondrial reference genome (GenBank No. V00654) using BWA-0.7.17 [27] with the “aln”algorithm. Post-alignment BAM files were coordinate-sorted by 5’mapping positions using the “sort” algorithm in SAMtools-0.1.19 [28], followed by elimination of library-derived PCR duplicates through the “rmdup” algorithm implemented in the same software package. A high-confidence mitochondrial consensus sequence was reconstructed using ANGSD-0.938 [29] with the “-doFasta 2”parameter. Genome-wide depth distribution and regional coverage biases were quantified via Qualimap-2.2.1 [30], with regions with low coverage excluded from downstream analyses. Terminal nucleotide misincorporation patterns were statistically characterized using MapDamage 2.0 [31] to authenticate ancient DNA authenticity and estimate post-mortem degradation levels.

### 2.5. Mitochondrial Phylogenetic Analysis

The 13 mitochondrial genome sequences obtained in this study were combined with 162 mitochondrial genome sequences (72 for *B. primigenius*, 76 for *Bos taurus*, 9 for *Bos indicus,* and 5 for *Bison bonasus*) [6,18,32,33,34,35] downloaded from the NCBI database as the dataset for phylogenetic analyses (Tables S2 and S3), where *B. bonasus* was used as the outgroup. The 175 sequences were aligned using MAFFT 7.505 [36] from the CIPRES website [37]. A Maximum likelihood (ML) tree was then constructed using IQ-TREE v2.2.2.6 [38].

BEAST v1.8.4 [39] was used to calculate the divergence time between the Northeastern Chinese aurochs and other lineages, and determine the phylogenetic position of Northeastern Chinese aurochs. The best-fitting nucleotide substitution model (GTR + I + G) was inferred by BIC in jModelTest 2.1.6 [40]. An evolutionary rate with 2.043 ± 0.099 × 10^−8^ base substitutions per nucleotide per year was employed as a prior mutation rate for BEAST analyses [19]. Median calibrated ages of the samples were employed to calibrate the evolutionary rate as prior in the tip-dating sets. Other parameter settings were as follows: strict molecular clock; Coalescent: constant size; Markov chain Monte Carlo (MCMC) for 260 million iterations [18]. We used Tracer v1.7 [41] to check the convergence of posterior parameters, and whether effective sample sizes (ESS) were above 200. We constructed a Maximum Clade Credibility (MCC) tree using TreeAnnotator v1.8.4 [39] and used FigTree v1.4.3 (http://tree.bio.ed.ac) to visualize and annotate the resulting tree. Molecular dating of samples without radiocarbon dates was performed in BEAST using the exact age of all dated samples as tip-date calibration. Other parameters were the same as before. Additionally, we validated the dating results using samples that had already undergone radiocarbon dating (Figure S6).

To infer the population dynamics of haplogroup C aurochs and Northeastern China aurochs, two Bayesian Skyline Plots (BSP) were created using BEAST (Tables S2 and S3). Except for the molecular clock rate (a mean of 2.7308 × 10^−8^ substitutions per site per year, 95% interval 2.4314 × 10^−8^–3.0509 × 10^−8^) and MCMC set to 30 million iterations, all other parameters were the same as before. Finally, the Bayesian Skyline Plots were output using Tracer v1.7 [41].

## 3. Result

### 3.1. Radiocarbon Dating and the Genomes of Aurochs

We dated six of the thirteen auroch samples, which ranged from 3734 to 10,833 cal BP (Figure S4 and Table S1). For samples without dating information, we performed molecular dating separately for each individual, resulting in dates ranging from 3475 to 77,118 years BP (Table S1). Among all samples, four (CADG444, CADG609, CADG634, CADG681) date to the Pleistocene, while the remaining nine samples date to the Holocene.

To map the data using different reference genomes from aurochs and taurine cattle (GenBank No. KF525852, GU985279, and V00654), the mitochondrial genome of taurine cattle (GenBank No. V00654) was selected as the reference sequence to avoid potential biases introduced by same-species reference preference (Table S4). This generated 347–29,926 unique reads with 1.23–126× average coverage (Tables S4 and S5), resulting in the reconstructions of 13 near-complete mitochondrial genomes. The results of mapDamage2 suggested significant DNA fragmentation and high proportions of base mismatches at the ends of the fragments, confirming that our DNA sequences are of ancient origin (Figures S2 and S3).

### 3.2. Phylogenetic Analyses of Mitochondrial Genomes

**Figure 1:**
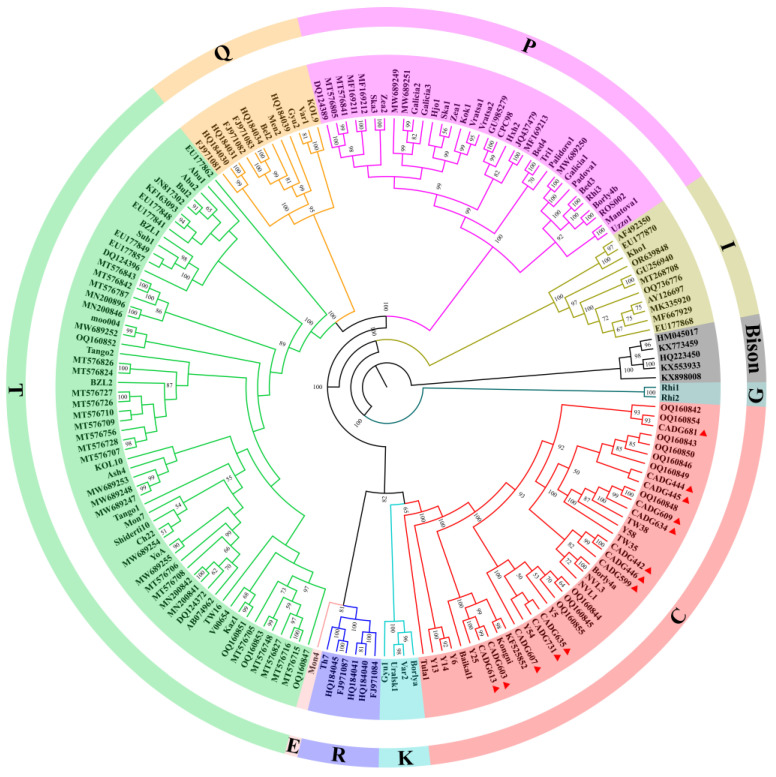
Maximum likelihood (ML) phylogenetic tree based on 175 complete mitogenome sequences (16,479 bp homologous sequences). Different colors indicate different haplogroups, the corresponding color haplogroups have been annotated in the figure. Red triangles indicate samples from this study; 1000 replicates were performed for each bootstrap value, and more than 50% of bootstrap values are shown in their neighborhood.

**Figure 2:**
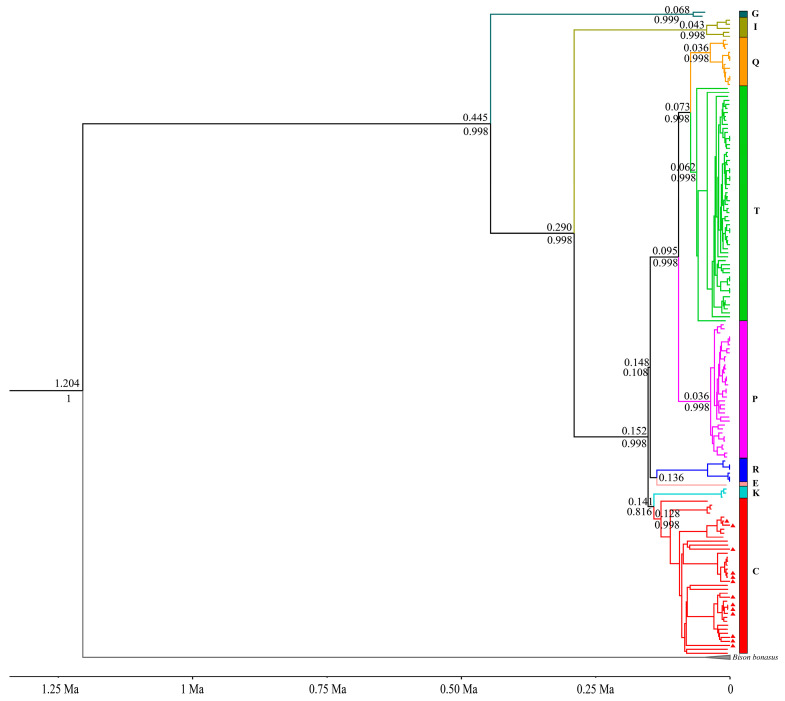
MCC tree in BEAST based on 16,479 bp homologous mitochondrial genome sequences. Different colors indicate different haplogroups, and our samples are indicated with red triangles. The numbers above nodes indicate divergence age, and the numbers below the nodes represent the Bayesian posterior probabilities.

ML and Bayesian trees were constructed based on nearly complete mitochondrial genomes using IQ-TREE and BEAST software, respectively. According to the topologies (Figure 1 and Figure 2), *B. primigenius*, *B. taurus*, and *B. indicus* can be divided into nine mitochondrial genetic lineages. The 13 samples in this study were clustered within haplogroup C clade and divided into several distinct subclades. Previous studies [18] and our Bayesian tree show that haplogroups R and E cluster with haplogroups P, T, and Q, but the ML tree shows that haplogroups R and E cluster within a clade with haplogroups C and K. Compared to the lower node prior in the Bayesian tree, the bootstrap value is higher in the ML tree.

Haplogroup G forms the sister lineage to all other haplogroups and separated at around 0.445 Ma (95% highest posterior density [HPD]: 0.388–0.509 Ma). Next was haplogroup I, i.e., the mitochondrial lineage of zebu-cattle that branched off at about 0.290 Ma (95% HPD: 0.249–0.335 Ma). Haplogroups C and K diverged from the other haplogroups at about 0.152 Ma (95% HPD: 0.132–0.174 Ma), and both separated at about 0.141 Ma (95% HPD: 0.122–0.161 Ma). Haplogroups P, T, and Q separated from haplogroups R and E at approximately 0.148 Ma (95% HPD: 0.128–0.168 Ma) (Figure 2 and Table 1).

### 3.3. Maternal Effective Population Size of Hyplogroup C

**Figure 3:**
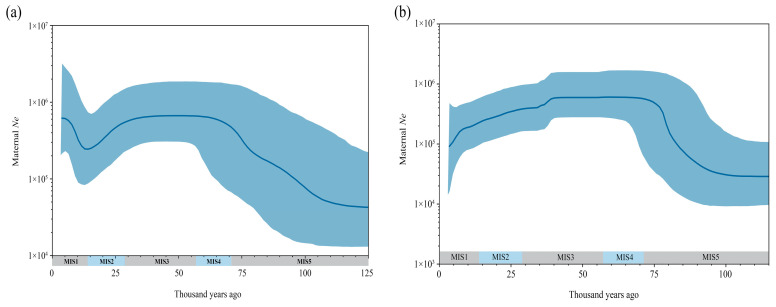
The Bayesian skyline plot is based on the complete mitogenome sequences of aurochs: (**a**,**b**) represent analyses of population dynamics in aurochs based on Haplogroup C lineages and Northeastern Chinese populations, respectively. The X-axis represents years before present (Ka), and the Y-axis indicates the effective maternal population size (Ne). The blue line is the median value, and the light blue shaded area represents the 95% confidence interval. Shading on the X-axis indicates Marine Isotope Stages (MIS).

The Bayesian skyline plot (BSP) constructed for aurochs individuals belonging to haplogroup C and the Northeastern China auroch population reveals fluctuations in the effective maternal population size over time (Figure 3). The haplogroup C lineage exhibited a significant demographic expansion during MIS5 (71–130 Ka) and MIS4 (57–71 Ka), followed by sustained population stability throughout the MIS3 (29–57 Ka). A pronounced decline occurred in MIS2 (14–29 Ka), with the subsequent recovery of effective population size observed in MIS1 (0–14 Ka) (Figure 3a). In contrast, the maternal effective population of aurochs in Northeastern China, after a significant increase during MIS5 stage, remained stable in MIS4 and the first half of MIS3, then began to decline in the second half of MIS3, culminating in its eventual extinction (Figure 3b).

## 4. Discussion

Both the ML tree and MCC tree indicate that our samples belong to the auroch haplogroup C. The Russian individual Tula1 occupies a basal position within haplogroup C, with the time to the most recent common ancestor (TMRCA) of haplogroup C estimated at 0.128 Ma (95% HPD: 0.111–0.147 Ma) (Figure 2 and Figure S5). Excluding this individual, the TMRCA for the remaining members of haplogroup C reduces to 0.111 Ma (95% HPD: 0.095–0.127 Ma) (Table 1). The inclusion of Tula1 substantially expands the temporal range of the haplogroup’s coalescence, and combined with its geographic provenance (the westernmost region of Russia), this raises questions regarding the taxonomic coherence of haplogroup C. Furthermore, while haplogroups G and I diverged earlier, other haplogroups underwent differentiation approximately 100,000 years ago. The diverging events of haplogroups P, T, and Q coincide with the internal diversification timeframe of haplogroup C. This temporal overlap suggests that subclades within haplogroup C correspond to the divergence scale typically used to define distinct haplogroups. Therefore, the definition of haplogroup C should be treated with caution, as well as the taxonomic attribution of the individual Tula1. Whether Tula1 should be classified as haplogroup C or named as a new haplogroup, as well as the previous classification of the clades of haplogroup C (which divided haplogroup C into the three clades of C1, C2, and C3) [15], needs to be further investigated and discussed. In addition, the positions of haplogroups R and E are inconsistent in the ML and Bayesian trees, but the likelihood of haplogroups R and E clustering into a clade with haplogroups C and K is higher because of the higher bootstrap value in the ML tree.

The population of haplogroup C experienced significant expansion during MIS 5 and MIS 4, remained stable throughout MIS 3, began to decline in MIS 2, and partially recovered during MIS 1. In contrast, the auroch population in Northeastern China expanded during MIS 5, maintained stability through MIS 4 and the early phase of MIS 3, but entered a sustained decline starting from the latter half of MIS 3. Comparative Bayesian skyline analyses reveal that the population dynamics of haplogroup C and the Northeastern Chinese aurochs were roughly the same from MIS5 to MIS2. However, a difference emerged during MIS 1: while haplogroup C exhibited recovery, the Northeastern Chinese population continued to decline, which may be related to the environmental shifts in Northeastern China. Since the onset of the Holocene (MIS 1), climatic conditions in this region deteriorated abruptly due to the weakening of the East Asian Summer Monsoon [42], marked by plummeting temperatures, reduced hydrological resources, and transformative vegetation changes [43,44,45]. Concurrently, anthropogenic pressures—including overhunting driven by the aurochs’ critical role as a source of dietary protein [46], raw materials for bone tool production [47], and ritual practices [11]—likely exacerbated population stress [33]. So, both climate adversity and human activities may have some impact on the decline of aurochs populations in Northeastern China.

**Figure 4:**
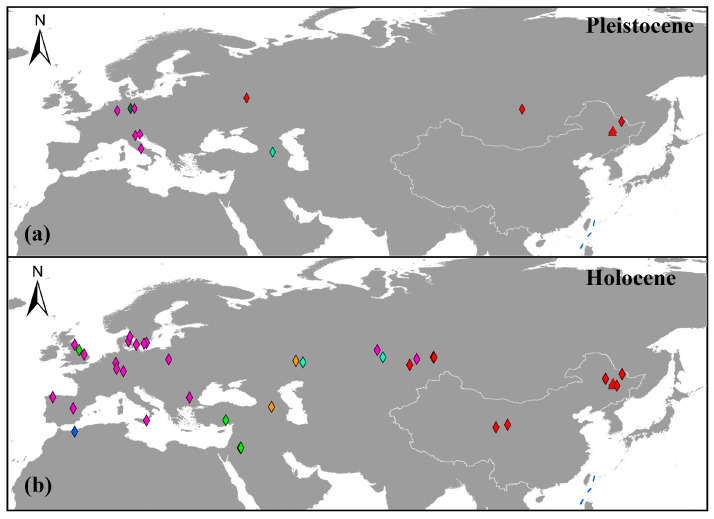
The geographical distribution of aurochs: (**a**,**b**) represent the distribution sites of aurochs during the Pleistocene and Holocene. Different colors represent different haplogroups, which is consistent with the description in the previous phylogenetic analysis. Red represents haplogroup C, green represents haplogroup T, orange represents haplogroup Q, magenta represents haplogroup P, blue represents haplogroup R, dark green represents haplogroup G, and light blue represents haplogroup K. The samples from this study are represented by red triangles.

Prior to extinction, Northeastern China appears to have played a key role in the persistence and diversity of aurochs. Our auroch samples are located in different subclades of haplogroup C and date from the Late Pleistocene to the Holocene. Notably, with the exception of five recently identified individuals from Russia and Kazakhstan, all known haplogroup C specimens originate exclusively from China (Figure 4). This geographic distribution suggests that Northeastern China may have sustained a significant auroch population throughout the Late Pleistocene and Holocene. The wealth of fossil evidence from this region further supports this conclusion [4,5,11,13,15,16,17,33,48]. In addition, this region supported a diverse Pleistocene megafauna community, including cave hyenas (*Crocuta crocuta*) [49], steppe bison (*Bison priscus*) [50], woolly rhinoceros (*Coelodonta antiquitatis*) [51], tigers (*Panthera tigris*) [52], and giant deer (*Sinomegaceros* spp.) [53], many of which harbored phylogenetically distinct ancient lineages. The prolonged coexistence and persistence of these species indicate that Northeastern China served as a critical survival site for Quaternary mammals. Consequently, this area represents a vital focal point for investigating evolutionary dynamics and adaptive strategies of Pleistocene megafauna under environmental stressors, particularly in the context of climatic oscillations and anthropogenic impacts.

In summary, this study conducted mitochondrial genome analyses on auroch specimens from Northeastern China. The results demonstrate that these individuals belong to haplogroup C. However, based on the divergence times between haplogroup C and other haplogroups, we argue that the current phylogenetic delineation of haplogroup C is overly broad, with certain subclades warranting reclassification as distinct haplogroups. Moreover, the slightly different population dynamics based on all haplogroups C and those based only on aurochs in Northeastern China provide evidence for different fates of populations carrying haplogroup C in different regions. Northeastern China has played an important role in protecting the persistence and genetic diversity of aurochs, while also promoting the survival of Quaternary mammals. Nevertheless, during the Holocene, synergistic pressures from climatic deterioration and intensified anthropogenic hunting caused the aurochs population decline and ultimately leads to extinction in Northeastern China.

## Figures and Tables

**Table 1:** Node ages of haplogroup divergences.

Tree Node	Median Node Age (BP)	Node Age 95% HPD (BP)	Node Prior
Bos/Bison	1,204,325	1,060,461–1,351,560	1
G/IKCREPQT	445,694	388,651–509,781	0.9981
G	68,514	59,737–78,107	0.9992
I/KCREPQT	290,170	249,661–335,306	0.9981
I	43,476	31,578–56,933	0.9981
CK/REPQT	152,505	132,706–174,699	0.9981
C/K	141,660	122,846–161,742	0.8165
C	128,485	111,552–147,478	0.9982
C-Tula1 *	111,174	95,690–127,359	0.9981
K	16,191	13,922–20,091	0.9991
RE/PQT	148,488	128,426–168,895	0.1081
R/E	136,181	114,341–157,965	0.9153
R	41,785	29,876–54,517	0.9983
P/TQ	95,767	80,455–112,706	0.9983
P	36,098	29,463–43,094	0.9983
T/Q	73,409	60,517–87,489	0.9984
T	61,968	50,783–74,595	0.998
Q	36,651	24,884–51,722	0.998

* denotes the common ancestor time of haplogroup C excluding individual Tula1.

## Data Availability

Thirteen ancient mitochondrial genomes can be found under the GenBank Accession codes PV019473-PV019481 and PV132380-PV132383. The address is as follows: GenBank www.ncbi.nlm.nih.gov/genbank, accessed on 28 January 2025.

## Supplementary Materials

The following supporting information can be downloaded at: https://www.mdpi.com/article/10.3390/genes16060639/s1, Table S1: Specific information of the samples; Table S2: Ancient genomic data information that was previously analyzed for this study; Table S3: Mitochondrial DNA that was previously analyzed for this study; Table S4: Results of mapping data against different reference sequences; Table S5: Raw data mapping statistics for *Bos taurus* (Mitochondrial reference: V00654.1); Table S6: Information of the three overlapping long range PCR (LR-PCR) primer pairs used in hybridization capture library construction; Figure S1: Photographs of part samples with a more complete appearance in this study; Figure S2: Mitochondrial fragment length distribution of partial samples analyzed in this study; Figure S3: Mitochondrial cytosine deamination frequency inferred from the partial auroch samples analyzed in this study; Figure S4: Radiocarbon dating of the six samples in this study; Figure S5: The MCC tree in BEAST is based on 16,479 bp homologous mitochondrial genome sequences. Different colors denote distinct haplogroups, corresponding to Figure 2. The red triangles represent our samples. The numbers above the nodes indicate divergence age; Figure S6: The molecular dating results of some radiocarbon dating samples. References [6,13,14,15,16,18,19,32,33,34,47,54,55,56,57,58,59,60,61,62,63,64,65,66,67,68,69,70,71,72] are added in the Supplementary file.
