# Supplementary material for: Revisiting Aurochs Haplogroup C: Paleogenomic Perspectives from Northeastern China
Source: Genes (Basel). 2025 May 27;16(6):639. doi: 10.3390/genes16060639 (PMC12192085; doi:10.3390/genes16060639)
Supplement: Supplementary file 1 [file genes-16-00639-s001.zip › genes-3651496-supplementary.pdf]

Table S1: Specific information of the samples.

Sample ID	Species	Skeletal element	Location	Calibrated radiocarbon age(cal BP)	Molecular dating(cal BP)
CADG442	*Bos primigenius*	rib	Zhaodong, Heilongjia, China	3734	
CADG444	*Bos primigenius*	metatarsus	Zhaodong, Heilongjia, China		55280
CADG445	*Bos primigenius*	rib	Zhaodong, Heilongjia, China		6854
CADG446	*Bos primigenius*	metacarpal bone	Zhaodong, Heilongjia, China	3803	
CADG599	*Bos primigenius*	mandible	Zhaodong, Heilongjia, China	3849	
CADG603	*Bos primigenius*	mandible	Zhaodong, Heilongjia, China	/	3475
CADG607	*Bos primigenius*	mandible	Zhaodong, Heilongjia, China	3853	
CADG609	*Bos primigenius*	mandible	Zhaodong, Heilongjia, China	/	77118
CADG613	*Bos primigenius*	mandible	Zhaodong, Heilongjia, China	10833	
CADG634	*Bos primigenius*	tooth	Qinggang, Heilongjia, China	/	20881
CADG635	*Bos primigenius*	tooth	Qinggang, Heilongjia, China	/	7798
CADG681	*Bos primigenius*	tooth	Zhaodong, Heilongjia, China	/	50070
CADG731	*Bos primigenius*	mandible	Zhaodong, Heilongjia, China	3840	

Table S2: Ancient genomic data information that was previously analyzed for this study.

Analysisn ID	Country	Analysis date given	Mitochondrial haplogroup	Species	Data source	Publication
Abu1	Israel	8000	T	*Bos primigenius*	PRJEB31621	Verdugo et al.2019[1]
Abu2	Israel	8000	T	*Bos primigenius*	PRJEB31621	Verdugo et al.2019[1]
Ash4	Israel	3100	T	*Bos taurus*	PRJEB31621	Verdugo et al.2019[1]
Baikal1	Russia	12198	C	*Bos primigenius*	PRJEB75467	Rossi et al.2024[2]
Bed3	Germany	11600	P	*Bos primigenius*	PRJEB74338	Erven et al.2024[3]
Bed4	Germany	11639	P	*Bos primigenius*	PRJEB75467	Rossi et al.2024[2]
Bel2	Serbia	6900	Q	*Bos taurus*	PRJEB31621	Verdugo et al.2019[1]
Borly4a	Kazakhstan	4946	C	*Bos primigenius*	PRJEB75467	Rossi et al.2024[2]
Borly4b	Kazakhstan	4932	P	*Bos primigenius*	PRJEB75467	Rossi et al.2024[2]
Borlya	Kazakhstan	6557	K	*Bos primigenius*	PRJEB75467	Rossi et al.2024[2]
Bul2	Uzbekistan	3800	T	*Bos taurus*	PRJEB75467	Rossi et al.2024[2]
BZL1	Russia	3990	T	*Bos taurus*	PRJEB75467	Rossi et al.2024[2]
BZL2	Russia	4022	T	*Bos taurus*	PRJEB75467	Rossi et al.2024[2]
Ch22	Turkey	7600	T	*Bos primigenius*	PRJEB31621	Verdugo et al.2019[1]
Galicia1	Spain	9250	P	*Bos primigenius*	PRJEB75467	Rossi et al.2024[2]
Galicia2	Spain	9217	P	*Bos primigenius*	PRJEB75467	Rossi et al.2024[2]
Galicia3	Spain	9257	P	*Bos primigenius*	PRJEB75467	Rossi et al.2024[2]
Gyu1	Armenia	13906	K	*Bos primigenius*	PRJEB75467	Rossi et al.2024[2]
Gyu2	Armenia	7136	Q	*Bos primigenius*	PRJEB31621	Verdugo et al.2019[1]
Hjo1	Denmark	8075	P	*Bos primigenius*	PRJEB75467	Rossi et al.2024[2]
Hxh2	Germany	5696	P	*Bos primigenius*	PRJEB75467	Rossi et al.2024[2]
Kaz1	Georgia	1000	T	*Bos taurus*	PRJEB31621	Verdugo et al.2019[1]
Kho1	Iran	1400	I	*Bos taurus*	PRJEB31621	Verdugo et al.2019[1]
Kok1	Uzbekistan	3500	P	*Bos taurus*	PRJEB31621	Verdugo et al.2019[1]
KOL9	Russia	4038	Q	*Bos taurus*	PRJEB75467	Rossi et al.2024[2]
KOL10	Russia	3907	T	*Bos taurus*	PRJEB75467	Rossi et al.2024[2]
Kongni	China	10665	C	*Bos primigenius*	/	Hou et al.2024[4]
Mantova1	Italy	13052	P	*Bos primigenius*	PRJEB75467	Rossi et al.2024[2]
Men2	Turkey	7924	Q	*Bos taurus*	PRJEB31621	Verdugo et al.2019[1]
Mon4	Turkmenistan	6371	E	*Bos taurus*	PRJEB75467	Rossi et al.2024[2]
Mon7	Turkmenistan	6804	T	*Bos taurus*	PRJEB75467	Rossi et al.2024[2]
moo004	Iberia	/	T	*Bos primigenius*	PRJEB63140	Günther et al.2025[5]
NVL1	Russia	5149	C	*Bos primigenius*	PRJEB75467	Rossi et al.2024[2]
NVL3	Russia	5171	C	*Bos primigenius*	PRJEB75467	Rossi et al.2024[2]
Padova1	Italy	14005	P	*Bos primigenius*	PRJEB75467	Rossi et al.2024[2]
Palidoro1	Italy	18017	P	*Bos primigenius*	PRJEB75467	Rossi et al.2024[2]
Rhi1	Germany	51080	G	*Bos primigenius*	PRJEB75467	Rossi et al.2024[2]
Rhi2	Germany	46087	G	*Bos primigenius*	PRJEB75467	Rossi et al.2024[2]

Rhi3	Germany	12390	P	*Bos primigenius*	PRJEB75467	Rossi et al.2024[2]
ROS002	Kazakhstan	5386	P	*Bos primigenius*	PRJEB75467	Rossi et al.2024[2]
Shiderti10	Kazakhstan	3804	T	*Bos taurus*	PRJEB75467	Rossi et al.2024[2]
Ska1	Sweden	9335	P	*Bos primigenius*	PRJEB75467	Rossi et al.2024[2]
Ska3	Sweden	9546	P	*Bos primigenius*	PRJEB75467	Rossi et al.2024[2]
Sub1	Turkey	8072	T	*Bos taurus*	PRJEB31621	Verdugo et al.2019[1]
Tango1	/	3954	T	*Bos primigenius*	PRJEB75467	Rossi et al.2024[2]
Tango2	/	4415	T	*Bos primigenius*	PRJEB75467	Rossi et al.2024[2]
Th7	Morocco	8828	R	*Bos primigenius*	PRJEB31621	Verdugo et al.2019[1]
Tri1	Germany	10060	P	*Bos primigenius*	PRJEB75467	Rossi et al.2024[2]
Tula1	Russia	41529	C	*Bos primigenius*	PRJEB75467	Rossi et al.2024[2]
TW16	China	3587	T	*Bos taurus*	PRJCA024258	Chen et al.2024[6]
TW35	China	3410	C	*Bos primigenius*	PRJCA024258	Chen et al.2024[6]
TW38	China	3744	C	*Bos primigenius*	PRJCA024258	Chen et al.2024[6]
Uralsk1	Kazakhstan	/	K	*Bos primigenius*	PRJEB75467	Rossi et al.2024[2]
Uzzo1	Italy	9486	P	*Bos primigenius*	PRJEB75467	Rossi et al.2024[2]
Var1	Russia	7497	Q	*Bos primigenius*	PRJEB75467	Rossi et al.2024[2]
Var2	Russia	7288	K	*Bos primigenius*	PRJEB75467	Rossi et al.2024[2]
Vratsa1	Bulgaria	6058	P	*Bos primigenius*	PRJEB75467	Rossi et al.2024[2]
Vratsa2	Bulgaria	6130	P	*Bos primigenius*	PRJEB75467	Rossi et al.2024[2]
Y5	China	3852	C	*Bos primigenius*	PRJNA781671	Hou et al.2024[4]
Y6	China	34217	C	*Bos primigenius*	PRJNA781671	Hou et al.2024[4]
Y13	China	37011	C	*Bos primigenius*	PRJNA781671	Hou et al.2024[4]
Y14	China	33162	C	*Bos primigenius*	PRJNA781671	Hou et al.2024[4]
Y25	China	10622	C	*Bos primigenius*	PRJNA781671	Hou et al.2024[4]
Y54	China	4115	C	*Bos primigenius*	PRJNA781671	Hou et al.2024[4]
Y58	China	4280	C	*Bos primigenius*	PRJNA781671	Hou et al.2024[4]
YoA	Britain	4710	T	*Bos primigenius*	PRJEB75467	Rossi et al.2024[2]
Zea1	Denmark	7302	P	*Bos primigenius*	PRJEB75467	Rossi et al.2024[2]
Zea2	Denmark	7296	P	*Bos primigenius*	PRJEB75467	Rossi et al.2024[2]

Table S3: Mitochondrial DNA that was previously analyzed for this study.

GenBank accession No.	Country	Age(yr BP)	Species	Publication
GU985279	Britain	6738	*Bos primigenius*	Edwards et al.2010[7]
KF525852	China	10660	*Bos primigenius*	Zhang et al.2013[8]
JQ437479	Poland	1500	*Bos primigenius*	Zeyland et al.2013[9]
MW689249	Spain	9295	*Bos primigenius*	Gurke et al.2021[10]
MW689250	Spain	9299	*Bos primigenius*	Gurke et al.2021[10]
MW689251	Spain	9216	*Bos primigenius*	Gurke et al.2021[10]
CPC98	Britain	6738	*Bos primigenius*	Park et al.2015[11]
MF169213	Denmark	550	*Bos primigenius*	Bro-Jørgensen et al.2018[12]
MF169212	Denmark	550	*Bos primigenius*	Bro-Jørgensen et al.2018[12]
MF169211	Denmark	650	*Bos primigenius*	Bro-Jørgensen et al.2018[12]
OQ160855	Honghe, China	3508	*Bos primigenius*	Zhang et al.2023[13]
OQ160854	Honghe, China	3508	*Bos primigenius*	Zhang et al.2023[13]
OQ160850	Honghe, China	3508	*Bos primigenius*	Zhang et al.2023[13]
OQ160849	Honghe, China	4250	*Bos primigenius*	Zhang et al.2023[13]
OQ160848	Honghe, China	4250	*Bos primigenius*	Zhang et al.2023[13]
OQ160846	Honghe, China	4250	*Bos primigenius*	Zhang et al.2023[13]
OQ160845	Honghe, China	4250	*Bos primigenius*	Zhang et al.2023[13]
OQ160844	Honghe, China	4250	*Bos primigenius*	Zhang et al.2023[13]
OQ160843	Honghe, China	4250	*Bos primigenius*	Zhang et al.2023[13]
OQ160842	Honghe, China	4250	*Bos primigenius*	Zhang et al.2023[13]
OQ160847	Honghe, China	4250	*Bos taurus*	Zhang et al.2023[13]
OQ160851	Honghe, China	3508	*Bos taurus*	Zhang et al.2023[13]
OQ160852	Honghe, China	3508	*Bos taurus*	Zhang et al.2023[13]
OQ160853	Honghe, China	3508	*Bos taurus*	Zhang et al.2023[13]
EU177849	Greece	modern	*Bos taurus*	Achilli et al.2008[14]
MT576705	Shimao, China	3900	*Bos taurus*	Xia et al.2021[15]
MT576706	Shimao, China	3900	*Bos taurus*	Xia et al.2021[15]
MT576707	Shimao, China	3900	*Bos taurus*	Xia et al.2021[15]
MT576708	Shimao, China	3900	*Bos taurus*	Xia et al.2021[15]
MT576709	Shimao, China	3900	*Bos taurus*	Xia et al.2021[15]
MT576710	Shimao, China	3900	*Bos taurus*	Xia et al.2021[15]
MT576715	Anxi, China	modern	*Bos taurus*	Xia et al.2021[15]
MT576716	Anxi, China	modern	*Bos taurus*	Xia et al.2021[15]
MT576726	Tibet, China	modern	*Bos taurus*	Xia et al.2021[15]
MT576727	Tibet, China	modern	*Bos taurus*	Xia et al.2021[15]

MT576728	Tibet, China	modern	*Bos taurus*	Xia et al.2021[15]
MT576748	Tibet, China	modern	*Bos taurus*	Xia et al.2021[15]
MT576756	Russia	modern	*Bos taurus*	Xia et al.2021[15]
MT576787	Japan	modern	*Bos taurus*	Xia et al.2021[15]
MT576824	Mongolia	modern	*Bos taurus*	Xia et al.2021[15]
MT576826	Mongolia	modern	*Bos taurus*	Xia et al.2021[15]
MT576827	Mongolia	modern	*Bos taurus*	Xia et al.2021[15]
MT576841	Yanbian, China	modern	*Bos taurus*	Xia et al.2021[15]
MT576842	Yanbian, China	modern	*Bos taurus*	Xia et al.2021[15]
MT576843	Yanbian, China	modern	*Bos taurus*	Xia et al.2021[15]
AB074962	Japan	modern	*Bos taurus*	Mannen et al.2003[16]
MN200841	Yunna, China	modern	*Bos taurus*	Xia et al.2019[17]
MN200842	Yunna, China	modern	*Bos taurus*	Xia et al.2019[17]
MN200846	Yunna, China	modern	*Bos taurus*	Xia et al.2019[17]
MN200896	Yunna, China	modern	*Bos taurus*	Xia et al.2019[17]
KF163093	South Africa	modern	*Bos taurus*	Horsburgh et al.2013[18]
EU177841	Italy	modern	*Bos taurus*	Achilli et al.2008[14]
EU177862	Italy	modern	*Bos taurus*	Achilli et al.2008[14]
EU177868	Iraq	modern	*Bos taurus*	Achilli et al.2008[14]
EU177848	Iraq	modern	*Bos taurus*	Achilli et al.2008[14]
EU177870	Iran	modern	*Bos taurus*	Achilli et al.2008[14]
EU177857	Iran	modern	*Bos taurus*	Achilli et al.2008[14]
FJ971081	Italy	modern	*Bos taurus*	Achilli et al.2009[19]
FJ971084	Italy	modern	*Bos taurus*	Achilli et al.2009[19]
FJ971087	Italy	modern	*Bos taurus*	Achilli et al.2009[19]
HQ184031	Italy	modern	*Bos taurus*	Bonfiglio et al.2010[20]
HQ184034	Italy	modern	*Bos taurus*	Bonfiglio et al.2010[20]
HQ184039	Italy	modern	*Bos taurus*	Bonfiglio et al.2010[20]
DQ124372	Korea	modern	*Bos taurus*	/
DQ124389	Korea	modern	*Bos taurus*	/
DQ124396	Korea	modern	*Bos taurus*	/
V00654	/	modern	*Bos taurus*	Anderson et al.1982[21]
MW689247	Spain	1143	*Bos taurus*	Gurke et al.2021[10]
MW689248	Spain	2535	*Bos taurus*	Gurke et al.2021[10]
MW689252	Spain	3592	*Bos taurus*	Gurke et al.2021[10]
MW689253	Spain	1481	*Bos taurus*	Gurke et al.2021[10]
MW689254	Spain	1233	*Bos taurus*	Gurke et al.2021[10]
MW689255	Spain	1125	*Bos taurus*	Gurke et al.2021[10]
HQ184040	Italy	modern	*Bos taurus*	Bonfiglio et al.2010[20]
HQ184041	Italy	modern	*Bos taurus*	Bonfiglio et al.2010[20]
HQ184045	Italy	modern	*Bos taurus*	Bonfiglio et al.2010[20]

HQ184030	Italy	modern	*Bos taurus*	Bonfiglio et al.2010[20]
FJ971082	Italy	modern	*Bos taurus*	Achilli et al.2009[19]
FJ971083	Italy	modern	*Bos taurus*	Achilli et al.2009[19]
MT576805	Korea	modern	*Bos taurus*	Xia et al.2021[15]
GU256940	China	modern	*Bos indicus*	/
AF492350	Germany	/	*Bos indicus*	Hiendleder et al.2008[22]
AY126697	India	modern	*Bos indicus*	/
OR639848	India	/	*Bos indicus*	Arya et al.2025[23]
OQ736776	India	/	*Bos indicus*	Chakraborty et al.2023[24]
OQ886063	India	/	*Bos indicus*	Chakraborty et al.2023[24]
MT268708	/	/	*Bos indicus*	/
MF667929	India	/	*Bos indicus*	/
JN817302	Ethiopia	/	*Bos indicus*	Bonfiglio et al.2012[25]
MK335920	India	/	*Bos indicus*	De et al.2019[26]
HQ223450	/	/	*Bison bonasus*	/
HM045017	/	/	*Bison bonasus*	Zeyland et al.2012[27]
KX553933	/	/	*Bison bonasus*	Węcek et al.2017[28]
KX773459	/	/	*Bison bonasus*	Onar et al.2017[29]
KX898008	/	/	*Bison bonasus*	Massilani et al.2016[30]

Table S4: Results of mapping data against different reference sequences.

Sample ID	Mitogenome reference					
	V00654		GU985279		KF525852	
	Mapped reads	Mean coverage (×)	Mapped reads	Mean coverage (×)	Mapped reads	Mean coverage (×)
CADG442	4031	17.1901	4035	17.1779	3772	15.8709
CADG444	506402	126.1352	507426	125.995	470696	113.4009
CADG445	627	1.2307	625	1.2282	622	1.1746
CADG446	3711	16.3187	3731	16.3772	3479	15.1726
CADG599	3210	8.4958	3244	8.5485	3127	8.1875
CADG603	3728	6.935	3761	6.9912	3686	6.7811
CADG607	6318	17.4738	6331	17.4865	5988	16.4761
CADG609	4330	8.1396	4340	8.1445	4172	7.8018
CADG613	4650	11.7569	4654	11.7828	4469	11.1764
CADG634	1356	3.6713	1355	3.6818	1329	3.5887
CADG635	4862	5.8802	4883	5.9024	4801	5.8004
CADG681	2185	5.0275	2186	5.0308	2106	4.7983
CADG731	6802	11.2668	6803	11.2768	6531	10.6096

Table S5: Rawdata mapping statistics for *Bos taurus* (Mitochondrial reference: V00654.1).

Samples	Mappable reads	Mapped reads	Unique mapped reads	Average fragment lengths	Mapped bp	Total length (bp)	Depth
CADG442	40956955	4031	3390	82.86	280837	16307	17.2219
CADG444	21018504	506402	29926	68.86	2060499	16336	126.132
CADG445	20296943	627	347	57.94	20104	10551	1.89339
CADG446	25500221	3711	3130	85.19	266598	16287	16.3678
CADG599	64435399	3210	2331	59.55	138793	16142	8.59559
CADG603	83647008	3728	2073	54.66	113302	16082	7.04439
CADG607	46640283	6318	4451	64.15	285477	16293	17.5215
CADG609	61406608	4330	2332	57.03	132973	16152	8.23158
CADG613	64776252	4650	3071	62.55	192074	16225	11.8367
CADG634	74498348	1356	1104	54.34	59979	15341	3.90006
CADG635	116648858	4862	2022	47.52	96068	15114	6.35244
CADG681	69001900	2185	1203	68.29	82134	15728	5.21486
CADG731	107103265	6802	2902	63.44	184066	16218	11.3474

Table S6: Information of the three overlapping long range PCR (LR-PCR) primer pairs used in hybridization capture library construction.

Primer Name	Length (bp)	Tm°C	GC%	Sequence (5'-3')	PCR product length (bp)
946_For	20	61	50	AGGACTTGGCGGTGCTTTAT	6932
7877_Rev	20	61.1	55	AGAGAGGGCACAGCTCATGA	
7577_For	20	62.1	55	TAAAGCCAGGGGAGCTACGA	5669
13425_Rev	20	62.7	60	GTAGGGAATCGGGGTTGTCC	
13092_For	20	59.5	50	ACATCTGTACCCACGCCTTT	4261
1013_Rev	21	59.7	42.9	AGGTTTATCGGGGTTTATCGA	

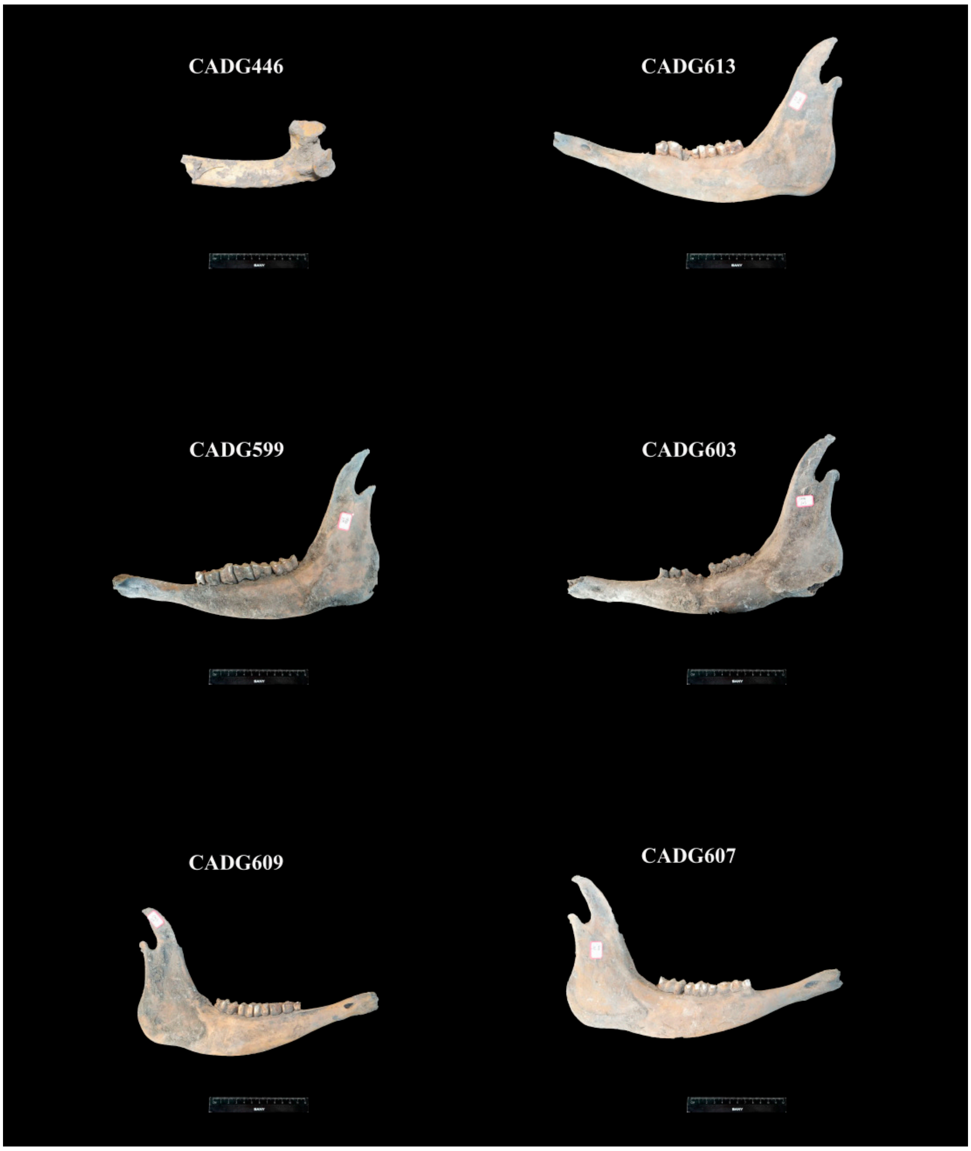

Figure S1: Photographs of well-conserved samples in this study.

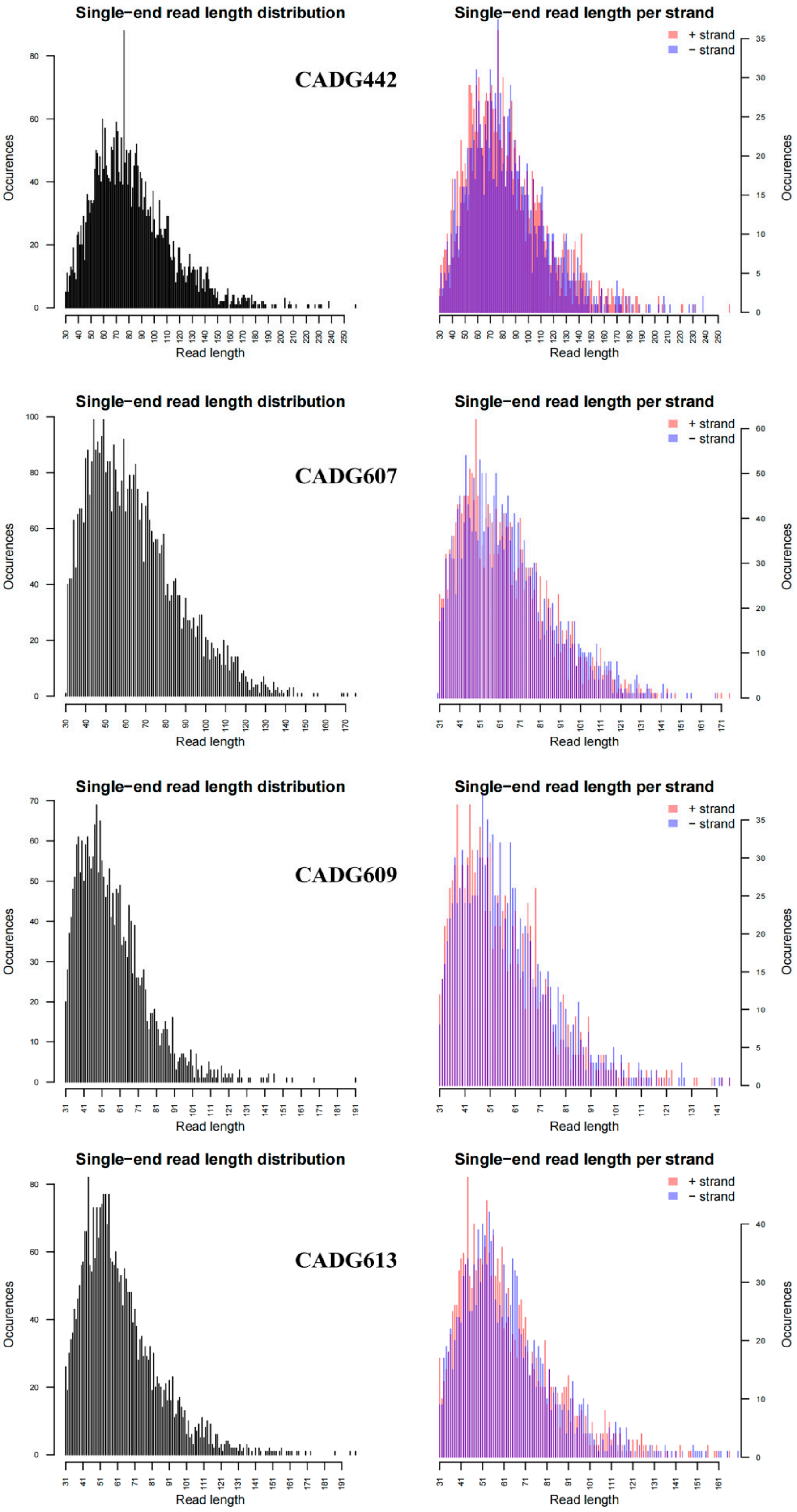

Figure S2: Mitochondrial fragment length distribution of partial samples analyzed in this study.

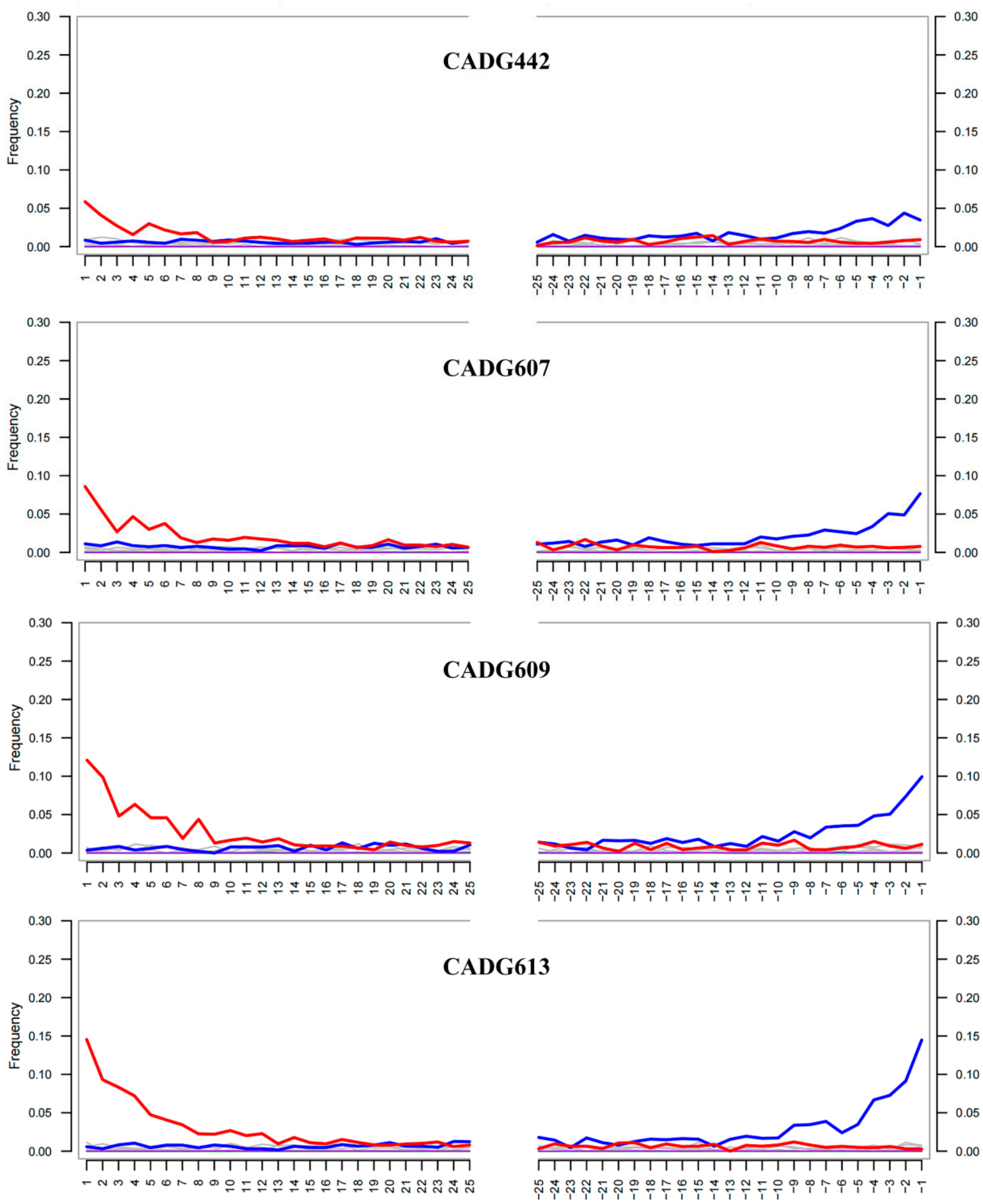

Figure S3: Mitochondrial cytosine deamination frequency inferred from the partial auroch samples analyzed in this study.

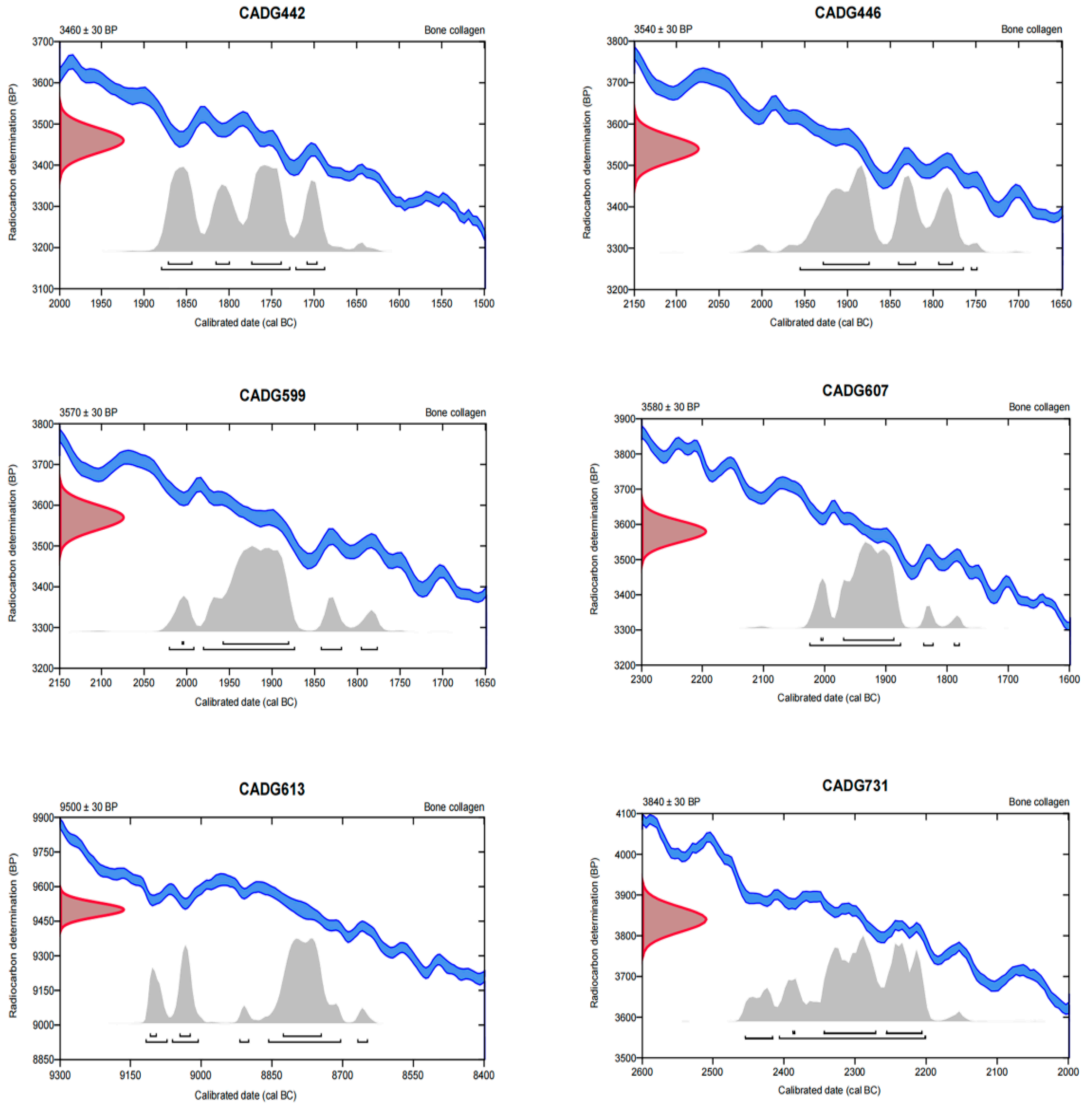

Figure S4: Radiocarbon dating of the six samples in this study.

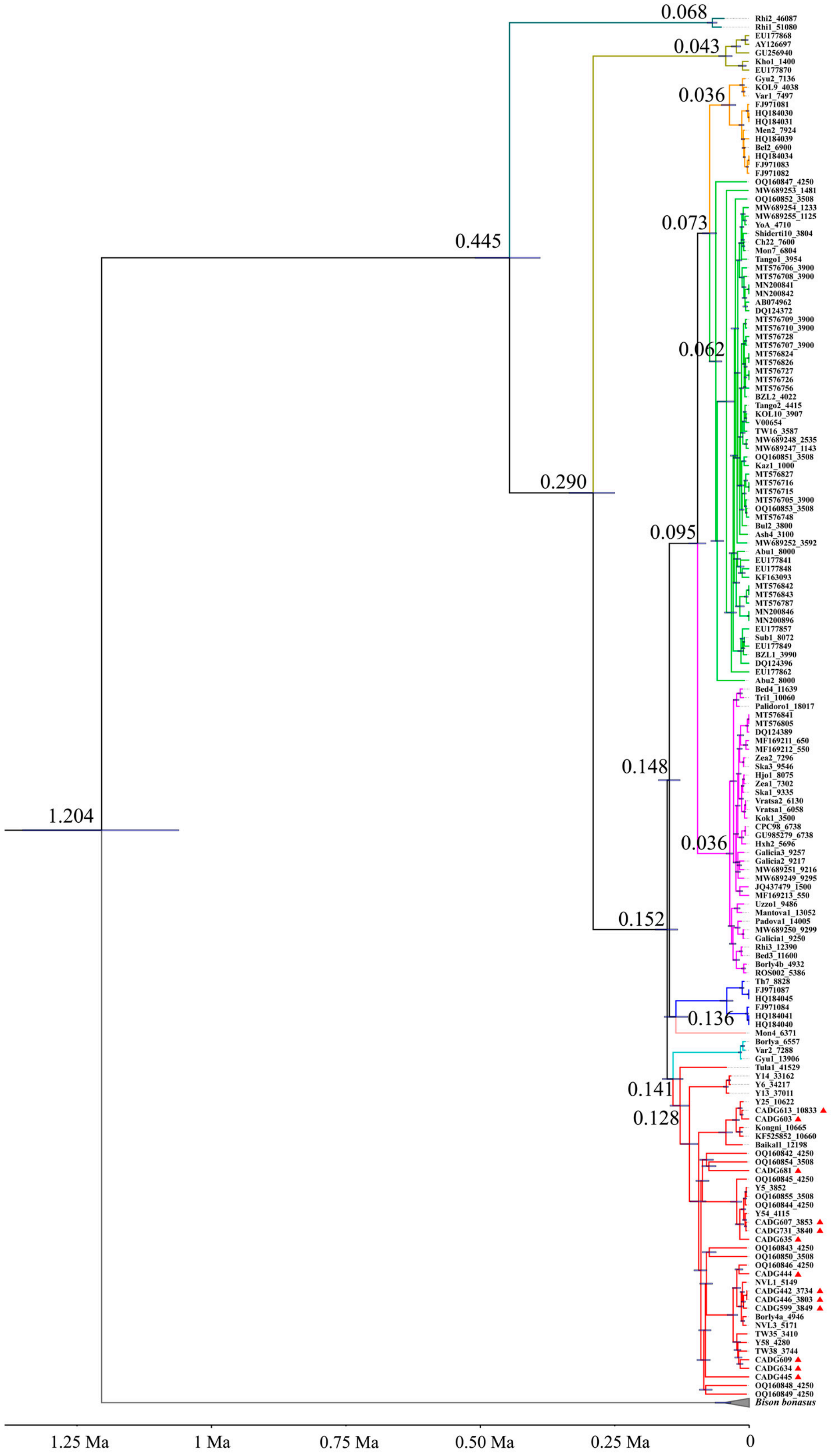

Figure S5: The MCC tree in BEAST is based on 16,479 bp homologous mitochondrial genome sequences. Different colors denote distinct haplogroups, corresponding to Figure 2. The red triangles represent our samples. The numbers above nodes indicate divergence age.

CADG442

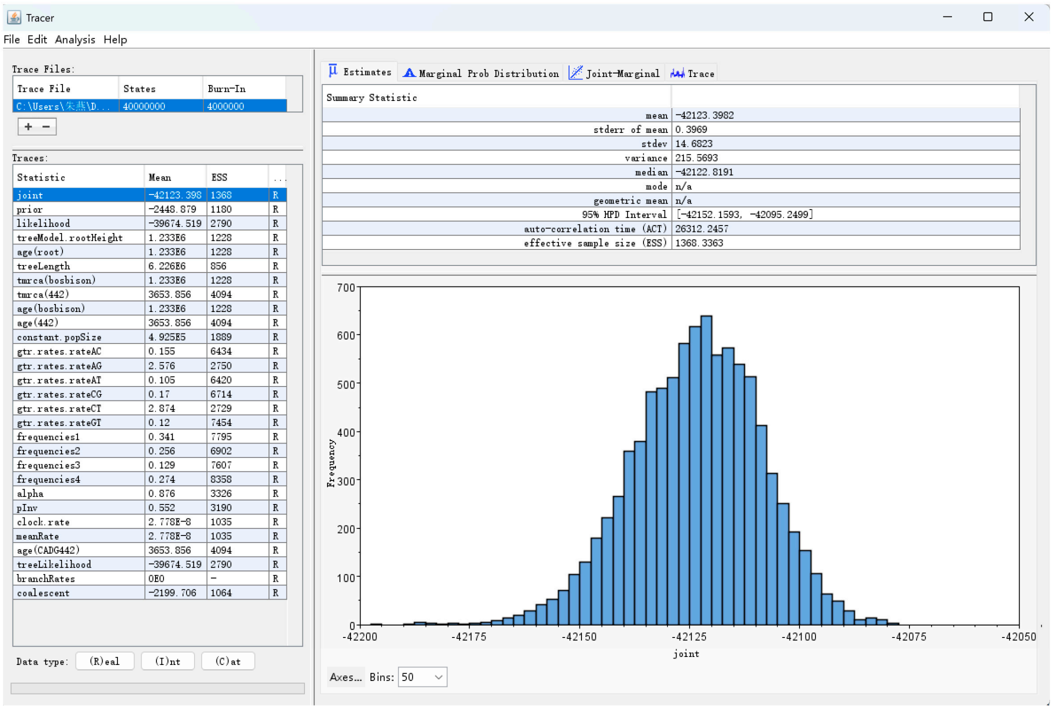

CADG446

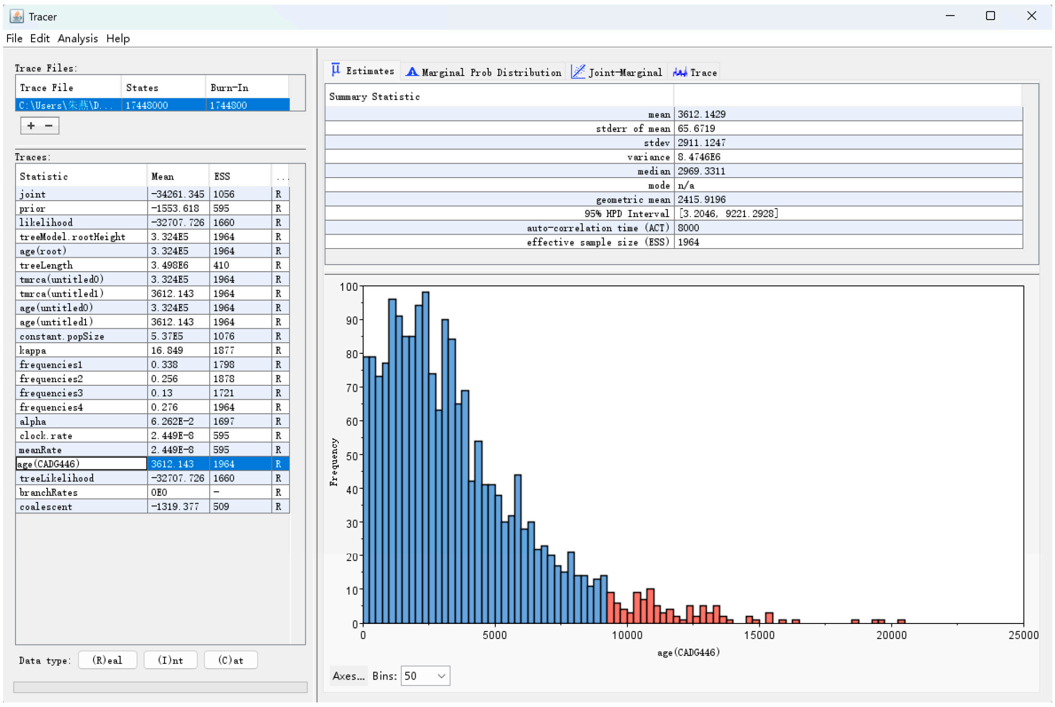

Figure S6: The molecular dating results of some radiocarbon dating samples.

References

1. Verdugo, M.P.; Mullin, V.E.; Scheu, A.; Mattiangeli, V.; Daly, K.G.; Maisano Delser, P.; Hare, A.J.; Burger, J.; Collins, M.J.; Kehati, R.; et al. Ancient cattle genomics, origins, and rapid turnover in the Fertile Crescent. *Science* **2019**, *365*, 173-176, doi:10.1126/science.aav1002.
2. Rossi, C.; Sinding, M.-H.S.; Mullin, V.E.; Scheu, A.; Erven, J.A.M.; Verdugo, M.P.; Daly, K.G.; Ciucani, M.M.; Mattiangeli, V.; Teasdale, M.D.; et al. The genomic natural history of the aurochs. *Nature* **2024**, *635*, 136-141, doi:10.1038/s41586-024-08112-6.
3. Erven, J.A.M.; Scheu, A.; Verdugo, M.P.; Cassidy, L.; Chen, N.B.; Gehlen, B.; Street, M.; Madsen, O.; Mullin, V.E. A high-coverage Mesolithic aurochs genome and effective leveraging of ancient cattle genomes using whole genome imputation. *Mol. Biol. Evol.* **2024**, *41*, msae076, doi:10.1093/molbev/msae076.
4. Hou, J.W.; Guan, X.W.; Xia, X.T.; Lyu, Y.; Liu, X.; Mazei, Y.; Xie, P.; Chang, F.Q.; Zhang, X.N.; Chen, J.L.; et al. Evolution and legacy of East Asian aurochs. *Sci Bull* **2024**, *69*, 3425-3433, doi:10.1016/j.scib.2024.09.016.
5. Günther, T.; Chisausky, J.; Galindo-Pellicena, M.Á.; Iriarte, E.; Gardyn, O.C.; Eusebi, P.G.; García-González, R.; Urena, I.; Moreno, M.; Alday, A.; et al. The genomic legacy of human management and sex-biased aurochs hybridization in Iberian cattle. *eLife* **2025**, *13*, RP93076, doi:10.7554/eLife.93076. .
6. Chen, S.G.; Ren, L.L.; Gao, Y.; Dong, G.H.; Sheng, G.L.; Han, J.L.; Liu, X.Y.; Chen, N.B.; Chen, F.H. Evidence of hybridization of cattle and aurochs on the Tibetan Plateau ~3750 years ago. *Sci Bull* **2024**, *69*, 2825-2828, doi:10.1016/j.scib.2024.06.035.
7. Edwards, C.J.; Magee, D.A.; Park, S.D.E.; McGettigan, P.A.; Lohan, A.J.; Murphy, A.; Finlay, E.K.; Shapiro, B.; Chamberlain, A.T.; Richards, M.B.; et al. A complete mitochondrial genome sequence from a mesolithic wild aurochs (*Bos primigenius*). *PLoS One* **2010**, *5*, e9255, doi:10.1371/journal.pone.0009255.
8. Zhang, H.C.; Paijmans, J.L.A.; Chang, F.Q.; Wu, X.H.; Chen, G.J.; Lei, C.Z.; Yang, X.J.; Wei, Z.Y.; Bradley, D.G.; Orlando, L.; et al. Morphological and genetic evidence for early Holocene cattle management in northeastern China. *Nat. Commun.* **2013**, *4*, 2755, doi:10.1038/ncomms3755.
9. Zeyland, J.; Wolko, Ł.; Bocianowski, J.; Szalata, M.; Słomski, R.; Dzieduszycki, A.M.; Ryba, M.; Przystałowska, H.; Lipiński, D. Complete mitochondrial genome of wild aurochs (*Bos primigenius*) reconstructed from ancient DNA. *Pol. J. Vet. Sci.* **2013**, *16*, 265-273, doi:10.2478/pjvs-2013-0037.
10. Gurke, M.; Vidal-Gorosquieta, A.; Pajimans, J.L.A.; Węcek, K.; Barlow, A.; González-Fortes, G.; Hartmann, S.; Grandal-d'Anglade, A.; Hofreiter, M. Insight into the introduction of domestic cattle and the process of Neolithization to the Spanish region Galicia by genetic evidence. *PLoS One* **2021**, *16*, e0249537, doi:10.1371/journal. pone.0269578.
11. Park, S.D.E.; Magee, D.A.; McGettigan, P.A.; Teasdale, M.D.; Edwards, C.J.; Lohan, A.J.; Murphy, A.; Braud, M.; Donoghue, M.T.; Liu, Y.; et al. Genome sequencing of the extinct Eurasian wild aurochs, *Bos primigenius*, illuminates the phylogeography and evolution of cattle. *Genome Biol.* **2015**, *16*, 234, doi:10.1186/s13059-015-0790-2.

12. Bro-Jørgensen, M.H.; Carøe, C.; Vieira, F.G.; Nestor, S.; Hallström, A.; Gregersen, K.M.; Etting, V.; Gilbert, M.T.P.; Sinding, M.-H.S. Ancient DNA analysis of Scandinavian medieval drinking horns and the horn of the last aurochs bull. *J. Archaeol. Sci.* **2018**, *99*, 47-54, doi:10.1016/j.jas.2018.09.001.
13. Zhang, N.F.; Liang, Q.Y.; Shao, X.Y.; Guo, Y.Q.; Wang, Y.D.; Wang, X.C.; Zhang, W.; Ning, C.; Cai, D.W. Ancient cattle DNA provides novel insight into the subsistence mode transition from the late Neolithic to Bronze Age in the Nenjiang River Basin. *J. Archaeol. Sci. Rep.* **2023**, *51*, 104136, doi:10.1016/j.jasrep.2023.104136.
14. Achilli, A.; Olivieri, A.; Pellecchia, M.; Uboldi, C.; Colli, L.; Al-Zahery, N.; Accetturo, M.; Pala, M.; Kashani, B.H.; Perego, U.A.; et al. Mitochondrial genomes of extinct aurochs survive in domestic cattle. *Curr. Biol.* **2008**, *18*, R157-R158, doi:10.1016/j.cub. 2008.01.019.
15. Xia, X.T.; Achilli, A.; Lenstra, J.A.; Tong, B.; Ma, Y.; Huang, Y.Z.; Han, J.L.; Sun, Z.Y.; Chen, H.; Lei, C.Z.; et al. Mitochondrial genomes from modern and ancient Turano-Mongolian cattle reveal an ancient diversity of taurine maternal lineages in East Asia. *Heredity* **2021**, *126*, 1000-1008, doi:10.1038/s41437-021-00428-7.
16. Mannen, H.; Morimoto, M.L.; Oyamat, K.; Mukai, F.; Tsuji, S. Identification of mitochondrial DNA substitutions related to meat quality in Japanese Black cattle. *J. Anim. Sci.* **2003**, *81*, 68-73, doi:10.2527/2003.81168x.
17. Xia, X.T.; Qu, K.X.; Li, F.Y.; Jia, P.; Chen, Q.M.; Chen, N.B.; Zhang, J.C.; Chen, H.; Huang, B.Z.; Lei, C.Z. Abundant genetic diversity of Yunling cattle based on mitochondrial genome. *Animals (Basel)* **2019**, *9*, 641, doi:10.3390/ani9090641.
18. Horsburgh, K.A.; Prost, S.; Gosling, A.; Stanton, J.A.; Rand, C.; Matisoo-Smith, E.A. The genetic diversity of the Nguni breed of African cattle (*Bos* spp.): complete mitochondrial genomes of haplogroup T1. *PLoS One* **2013**, *8*, e71956, doi:10.1371/journal.pone. 0071956.
19. Achilli, A.; Bonfiglio, S.; Olivieri, A.; Malusà, A.; Pala, M.; Kashani, B.H.; Perego, U.A.; Ajmone-Marsan, P.; Liotta, L.; Semino, O.; et al. The multifaceted origin of taurine cattle reflected by the mitochondrial genome. *PLoS One* **2009**, *4*, doi:10.1371/journal.pone. 0005753.
20. Bonfiglio, S.; Achilli, A.; Olivieri, A.; Negrini, R.; Colli, L.; Liotta, L.; Ajmone-Marsan, P.; Torroni, A.; Ferretti, L. The enigmatic origin of bovine mtDNA haplogroup R: sporadic interbreeding or an independent event of *Bos primigenius* domestication in Italy? *PLoS One* **2010**, *5*, e15760, doi:10.1371/journal.pone.0015760.
21. Anderson, S.; De Bruijn, M.H.; Coulson, A.R.; Eperon, I.C.; Sanger, F.; Young, I.G. Complete sequence of bovine mitochondrial DNA. Conserved features of the mammalian mitochondrial genome. *J. Mol. Biol.* **1982**, *156*, 683-717, doi:10.1016/0022-2836(82) 90137-1.
22. Hiendleder, S.; Lewalski, H.; Janke, A. Complete mitochondrial genomes of *Bos taurus* and *Bos indicus* provide new insights into intra-species variation, taxonomy and domestication. *Cytogenet Genome Res.* **2008**, *120*, 150-156, doi:10.1159/000118756.
23. Arya, M.; Ghosh, A.; Tyagi, K.; Tyagi, I.; Bisht, S.S.; Kumar, V. Characterization of complete mitochondrial genome of Badri breed of *Bos indicus* (Bovidae: Bovinae): selection pressure and comparative analysis. *Biochem. Genet.* **2025**, *63*, 43-66, doi:10.1007/s10528-024-10691-y.

24. Chakraborty, A.; Bisht, M.S.; Saxena, R.; Mahajan, S.; Pulikkan, J.; Sharma, V.K. Genome sequencing and de novo and reference-based genome assemblies of *Bos indicus* breeds. *Genes Genom.* **2023**, *45*, 1399-1408, doi:10.1007/s13258-023-01401-w.
25. Bonfiglio, S.; Ginja, C.; De Gaetano, A.; Achilli, A.; Olivieri, A.; Colli, L.; Tesfaye, K.; Agha, S.H.; Gama, L.T.; Cattonaro, F.; et al. Origin and spread of *Bos taurus*: new clues from mitochondrial genomes belonging to haplogroup T1. *PLoS One* **2012**, *7*, e38601, doi:10.1371/journal.pone.0038601.
26. De, A.K.; Muthiyan, R.; George, Z.; Ponraj, P.; Malakar, D.; Kundu, A.; Sunder, J.; Bhattacharya, D. Complete mitochondrial genome of Trinket cattle, a Danish colonial leftover. *Mitochondrial DNA. Part B. Resources* **2019**, *4*, 2053-2054, doi:10.1080/23802359.2019.1618208.
27. Zeyland, J.; Wolko, L.; Lipinski, D.; Wozniak, A.; Nowak, A.; Szalata, M.; Bocianowski, J.; Slomski, R. Tracking of wisent-bison-yak mitochondrial evolution. *J. Appl. Genet.* **2012**, *53*, 317-322, doi:10.1007/s13353-012-0090-4.
28. Wecek, K.; Hartmann, S.; Paijmans, J.L.A.; Taron, U.; Xenikoudakis, G.; Cahill, J.A.; Heintzman, P.D.; Shapiro, B.; Baryshnikov, G.; Bunevich, A.N.; et al. Complex admixture preceded and followed the extinction of wisent in the wild. *Mol. Biol. Evol.* **2017**, *34*, 598-612, doi:10.1093/molbev/msw254.
29. Onar, V.; Soubrier, J.; Toker, N.Y.; Loenen, A.V.; Llamas, B.; Siddiq, A.B.; Pasicka, E.; Tokarska, M. Did the historical range of the European bison (*Bison bonasus* L.) extend further south?—a new finding from the Yenikapı Metro and Marmaray excavation, Turkey. *Mamm. Res.* **2017**, *62*, 103–109, doi:10.1007/s13364-016-0299-4.
30. Massilani, D.; Guimaraes, S.; Brugal, J.P.; Bennett, E.A.; Tokarska, M.; Arbogast, R.M.; Baryshnikov, G.; Boeskorov, G.; Castel, J.C.; Davydov, S.; et al. Past climate changes, population dynamics and the origin of Bison in Europe. *BMC Biol.* **2016**, *14*, 93, doi:10.1186/s12915-016-0317-7.
